# Prevalence, incidence, repair rate, and morbidity of groin hernias in Sierra Leone: cross-sectional household study

**DOI:** 10.1093/bjsopen/zrac158

**Published:** 2023-01-19

**Authors:** Karel C Lindenbergh, Alex J van Duinen, Johan G Ahlbäck, Joseph Kamoh, Silleh Bah, Thomas Ashley, Jenny Löfgren, Martin P Grobusch, Osman Sankoh, Håkon A Bolkan, PRESSCO 2020 study group

**Affiliations:** VUmc School of Medical Sciences, Amsterdam, The Netherlands; Department of Public Health and Nursing, Norwegian University of Science and Technology, Trondheim, Norway; Department of Surgery, St. Olavs Hospital, Trondheim University Hospital, Trondheim, Norway; CapaCare, Norway, Sierra Leone, The Netherlands; Department of Surgery, St. Olavs Hospital, Trondheim University Hospital, Trondheim, Norway; CapaCare, Norway, Sierra Leone, The Netherlands; Statistics Sierra Leone, Tower Hill, Freetown, Sierra Leone; CapaCare, Norway, Sierra Leone, The Netherlands; Kamakwie Wesleyan Hospital, Kamakwie, Sierra Leone; Department of Molecular Medicine and Surgery, Karolinska Institutet, Stockholm, Sweden; Masanga Medical Research Unit, Masanga, Sierra Leone; Centre of Tropical Medicine and Travel Medicine, Amsterdam University Medical Centres, University of Amsterdam, Amsterdam, The Netherlands; Institute of Tropical Medicine, University of Tubingen, Tubingen, Germany; Centre de Recherches Medicales en Lambarene (CERMEL), Lambarene, Gabon; Institute of Infectious Diseases and Molecular Medicine (IDM), University of Cape Town, Cape Town, South Africa; Statistics Sierra Leone, Tower Hill, Freetown, Sierra Leone; School of Public Health, Faculty of Health Sciences, University of Witwatersrand, Johannesburg, South Africa; Heidelberg Institute of Global Health, University of Heidelberg Medical School, Heidelberg, Germany; Department of Public Health and Nursing, Norwegian University of Science and Technology, Trondheim, Norway; Department of Surgery, St. Olavs Hospital, Trondheim University Hospital, Trondheim, Norway; CapaCare, Norway, Sierra Leone, The Netherlands

## Abstract

**Background:**

Knowledge about the prevalence of groin hernias in sub-Saharan Africa is limited. Previous studies have demonstrated a higher incidence of the condition than the annual repair rate. This study aimed to investigate prevalence, incidence, annual repair rate, morbidity, and health-seeking behaviour of persons with groin hernias in Sierra Leone.

**Methods:**

This population-based, cross-sectional household survey on groin hernias in Sierra Leone was part of the Prevalence Study on Surgical Conditions 2020 (PRESSCO 2020). Those who indicated possible groin hernia were asked problem-specific questions and underwent physical examination to confirm or exclude the diagnosis.

**Results:**

3626 study participants were interviewed. The prevalence of untreated groin hernia was 1.1 per cent (95 per cent c.i. 0.8 to 1.5 per cent), whereas the prevalence of untreated and treated groin hernia was 2.5 per cent (95 per cent c.i. 2.0 to 3.0 per cent). The proportion of recurrence was 13.1 per cent. An incidence of 389 (95 per cent c.i. 213 to 652) groin hernia cases per 100 000 people per year was identified, while a population-based annual hernia repair rate estimation was 470 (95 per cent c.i. 350 to 620) per 100 000 people. Out of 39 participants with groin hernia, non-ignorable pain was reported by eight and 27 reported financial shortcomings as a reason for not seeking healthcare.

**Conclusions:**

Groin hernias are common in Sierra Leone and although the repair rate might match the incidence, the existing backlog of untreated hernias is likely to remain. It may be possible to reduce the number of recurrences through improved management. Measures to reduce financial barriers to treatment seem crucial to improve the health of people with groin hernias in Sierra Leone.

## Teaser

The prevalence of groin hernia in Sierra Leone is lower than previously reported in sub-Saharan Africa. The repair rate of groin hernia in Sierra Leone might keep up with the incidence of the condition. Insufficient treatment access for the rural population and financial barriers contribute to maintaining a backlog of untreated groin hernias.

## Introduction

Groin hernia repair is one of the most commonly performed surgical procedures worldwide, with an estimated volume of 20 million surgeries annually. The lifetime risk for having a groin hernia repair is 27.2 per cent for males and 2.6 per cent for females in high-income countries^1^. The estimated prevalence of groin hernia in adults, including women, in sub-Saharan Africa (SSA) is 3.2 to 5.4 per cent^2–4^. Two population-based studies from Uganda and Ghana including adult men only, demonstrated a prevalence of untreated groin hernia of 6.6 and 10.8 per cent respectively^5,6^. Most groin hernia studies from SSA focus on men^2–6^ and are based on self-reporting rather than physical examination^2–4^ and there are few prevalence estimations among women and children^7^. Two studies from Sierra Leone and Nepal found a prevalence of groin mass suspicious for hernia among women of 2.2 and 0.6 per cent respectively^3,8^. A study from Taiwan found a prevalence of groin hernia of 6.6 and 0.7 per cent for male and female children respectively and a recently published study from Uganda showed an overall prevalence of 1.4 per cent among children^9,10^. Groin hernia in women and children deserve particular attention due to a higher risk of complications compared with men^7,11^.

The incidence of groin hernia in SSA is underexplored^4^. Instead, hernia repair rates have been investigated. Previous studies from SSA have estimated the annual groin hernia repair rate to between 17 and 86 per 100 000 population^5,12–14^, compared with 140–240 per 100 000 in the European Union in 2018 and 275 per 100 000 in the USA in 2003^15,16^. The minimum required amount of inguinal hernia repairs in Eastern Africa was estimated at 205 per 100 000 people, which is in line with an estimation from Ghana of 210 per 100 000^4,17^. The low rate of herniorrhaphies in SSA countries indicates an unmet need for groin hernia surgeries, leading to avoidable ill health, death and economic loss^18^.

Improving access to groin hernia repair in SSA is essential in reaching universal health coverage. To reduce the burden of untreated groin hernias, better insight in the epidemiology and the reasons for failing to seek healthcare is needed, so that effective interventions can be planned^19^. The surgical landscape in Sierra Leone has been changing over time, for example by the introduction of task-sharing programmes, which has become a considerable part of the surgical workforce in the country^20^. It is important to monitor the development of surgical activities and local data may help to organize surgical services and lead health authorities in a sustainable direction. The aim of this study was to facilitate ongoing and future groin hernia management in Sierra Leone by determining the prevalence, incidence, and repair rate of groin hernia among men, women, and children, and by assessing health-seeking behaviour and impact of life associated with the condition.

## Methods

### Setting

Sierra Leone is a country in West Africa with 7.8 million inhabitants ranking 182 out of the 189 countries at the United Nations Development Index^21,22^. The life expectancy at birth of 54.7 years and other key health indicators are among the poorest worldwide^23^.

### Study design

This study was nested within the Prevalence Study on Surgical Conditions 2020 (PRESSCO 2020), a population-based, cross-sectional household survey on surgical and maternal conditions in Sierra Leone^24^. PRESSCO 2020 is a modified version of the Surgeons OverSeas Assessment of Surgical Need (SOSAS) tool that evaluated the prevalence of surgical conditions in Sierra Leone in 2012^25^. PRESSCO 2020 added several topics to the survey of SOSAS, including, among other things, additional questions and physical examination by a surgical provider of participants with a groin mass. This study was a secondary analysis of the data of PRESSCO 2020.

### Selection of study participants

Statistics Sierra Leone sampled 75 out of 9671 enumeration areas (EAs). Sampling weights and stratification were used to adjust for population size and density. From the 75 EAs, a total of 1875 households (25 households per EA) were randomly selected. Two randomly selected members in each household were approached for interview. Participants that remained absent after three repeated visits, were replaced by another randomly selected household member. To assess the hernia repair rate, the head of the household was asked if anyone currently living in the household had undergone a hernia repair in the past 12 months.

### Definitions

Groin hernia was diagnosed if there was a bulge in the groin with characteristics of groin hernia such as a positive cough reflex and/or reducibility by manual manipulation and the trained surgical provider excluded other causes. Groin hernia included both inguinal and femoral hernia. A treated groin hernia was defined as a groin hernia reported by the study participant and an observed surgical scar in the groin. A recurrent groin hernia included participants who reported having been operated on twice for hernia in the same groin as a scar presentation, and participants reporting past groin hernia surgery and ipsilaterally presented both a scar and a groin hernia during examination. Participants below 18 years of age were defined as children.

### Data collection

Interviews were performed by research nurses, surgical trained Community Health Officers (CHOs) and staff from Statistics Sierra Leone. A 1-week training session about all aspects of PRESSCO 2020 was provided to the enumerators. All participants were interviewed using a questionnaire, of which 19 nominal and ordinal questions were about health problems in the groin. The questionnaire was written in English, which is the official language in Sierra Leone. During the 1-week training session, attention was drawn to how to translate the questions to Krio which, besides English, is the most common language in Sierra Leone. It was decided to not translate the questionnaire to Krio or any other of the multiple languages used in Sierra Leone, as most enumerators only spoke three or four of the used languages in Sierra Leone. Participants reporting current or previous groin pathology received additional questions and underwent a physical examination carried out by third-year surgical CHO trainees, trained to independently diagnose and surgically treat common surgical conditions, including groin hernias^20^. The standardized physical examination included inspection of the groin mass and/or surgical scar, and palpation of the groin area. The surgical CHO categorized the swelling of the groin among the most common conditions in the groin and scrotum either as hernia, lipoma, lymphadenopathy, or hydrocele. Data were collected and managed using the electronic data collection tool REDCap hosted at Julius Center for Health Sciences and Primary Care (Utrecht, The Netherlands)^26,27^. All data were cross-checked at two levels; first within the EA by designated team members, and second, daily after uploading to the cloud-based server.

### Statistical analysis

Statistical analyses were performed with Stata® 17.1 (StataCorp, College Station, TX, USA). Prevalence and incidence are presented in percentages with a 95 per cent confidence interval (c.i.). Fisher’s exact test was used to compare categorical data.

### Consent and ethical considerations

All included participants (and their parents or guardians below 18 years of age) received oral (where possible in their local language) and written (in English) explanations about the study and were included after having given informed consent. Contact details of the research team were provided for participants to ask questions. Withdrawal of consent was possible both during and after the interview and examination. If the enumerators identified a participant or a relative of the participant in need of medical care, the CHO made a referral letter to the nearest relevant healthcare facility. Those who received a referral letter were not followed up as the capacity of PRESSCO 2020 was not dimensioned for this. After review of the study protocol by the Scientific Review Committee of the Masanga Medical Research Unit, ethical approval was granted by the Sierra Leone Ethics and Scientific Review Committee and the Norwegian Regional Committee for Medical and Health Research Ethics (REC 2019/31932). The study was registered at ISRCTN with the registration code ISRCTN12353489.

## Results

**Fig. 1:**
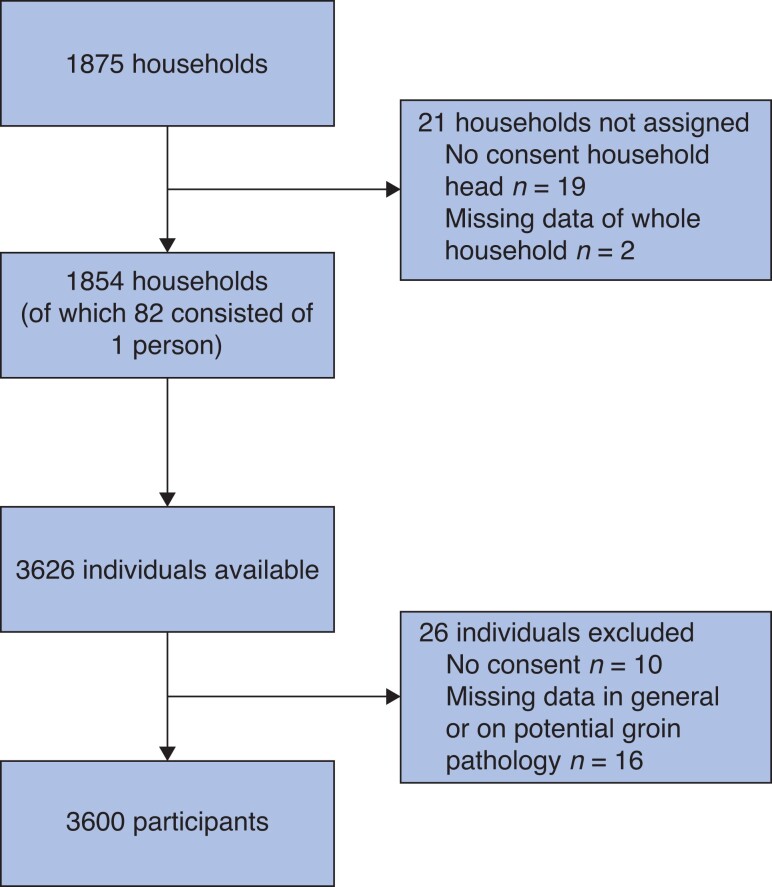
Flow chart of selection of study participants

Between October 2019 and March 2020, 3626 study participants were interviewed. Of all participants, 26 (0.7 per cent) were excluded due to lack of consent (10) or missing data (16) (*Fig. 1*). Half of the participants had not completed primary school, and the majority (67.4 per cent) lived in rural areas (*Table 1*).

**Table 1:** Characteristics of the study participants and prevalence for children, women, and men distributed by age

	Characteristics of study population	Untreated groin hernia	Treated groin hernia	No groin hernia
	*n* = 3600	*n* = 39 (1.1)	*n* = 61 (1.7)	*n* = 3511 (97.5)
				
**Age (years), mean (95% c.i.)**	24.7 (24.0, 25.3)	44.9 (37.7, 52.2)	52.7 (47.7, 57.8)	24.0 (23.4, 24.7)
**Children, age (years)**	1603 (44.5)	5 (12.8)	3 (4.9)	1595 (45.4)
0–4	511 (14.2)	1 (2.6)	0	510 (14.5)
5–17	1092 (30.3)	4 (10.3)	3 (4.9)	1085 (30.9)
**Women, age (years)**	1098 (30.5)	2 (5.1)	3 (4.9)	1093 (31.1)
18–39	720 (20.0)	1 (2.6)	1 (1.6)	718 (20.5)
40–59	254 (7.1)	1 (2.6)	1 (1.6)	252 (7.2)
60+	124 (3.5)	0	1 (1.6)	123 (3.5)
**Men, age (years)**	880 (24.4)	32 (82.1)	55 (90.2)	804 (22.9)
18–39	464 (13.0)	9 (23.1)	10 (16.4)	446 (12.7)
40–59	270 (7.1)	11 (28.2)	19 (31.1)	244 (6.9)
60+	146 (4.1)	12 (30.8)	26 (42.6)	114 (3.2)
Missing	19 (0.5)	0	0	19 (0.5)
**Highest education**				
Did not finish primary school	1797 (49.9)	24 (61.5)	32 (52.5)	1748 (49.8)
Primary school	908 (25.2)	5 (12.8)	11 (18.0)	893 (25.4)
Secondary school	735 (20.4)	7 (18.0)	12 (19.7)	717 (20.4)
Tertiary school or higher	143 (4.0)	3 (7.7)	6 (9.8)	136 (3.9)
Missing	17 (0.5)	0	0	17 (0.5)
**Occupation**				
Farming	898 (24.9)	26 (66.7)	35 (57.4)	846 (24.1)
Non-farming	1193 (33.1)	6 (15.4)	18 (29.5)	1171 (33.4)
Unemployed or attending school	1492 (41.4)	7 (17.9)	8 (13.1)	1477 (42.1)
Missing	17 (0.5)	0	0	17 (0.5)
**Residence**				
Rural	2425 (67.4)	34 (87.2)	46 (75.4)	2354 (67.0)
Urban	1175 (32.6)	5 (12.8)	15 (24.6)	1157 (33.0)

Values are *n* (%) unless otherwise indicated. Participants with recurrent (*n* = 8) or contralateral (*n* = 3) hernia are counted in both the categories for untreated and treated groin hernia.

### Prevalence and recurrence

Overall, 1.1 per cent (39, 95 per cent c.i. 0.8 to 1.5 per cent) of all participants presented with an untreated groin hernia, while 1.7 per cent (61, 95 per cent c.i. 1.3 to 2.2 per cent) had undergone groin hernia surgery. Corresponding numbers for men were 3.6 per cent (32) and 6.3 per cent (55), for women 0.2 per cent (2) and 0.3 per cent (3), and for children 0.3 per cent (5) and 0.2 per cent (3) respectively. The combined prevalence of treated and untreated groin hernia was 2.5 per cent (89, 95 per cent c.i. 2.0 to 3.0 per cent) overall, and for men, women, and children, 8.6 per cent (76), 0.5 per cent (5) and 0.3 per cent (8) respectively. No groin hernias were found in female children. Of men that reported previous hernia surgery and who had a scar (55), evidence of a recurrence was found in 14.5 per cent (8, 95 per cent c.i. 6.5 to 26.7 per cent). No recurrent groin hernias were found in women or children. Participants with an untreated groin hernia (39), were most often men (32), farmers (26), and living in rural areas (34). Also, they had a higher mean age than study participants with no reported groin hernia (44.9 *versus* 24.0 years). The prevalence of untreated groin hernia among men in rural areas was 4.8 per cent, compared with 1.1 per cent in urban areas (*P* = 0.007).

### Incidence and repair rate

Of all participants, 14 (0.4 per cent) reported they had developed their groin hernia within the last 12 months, which corresponds to an incidence of 389 (95 per cent c.i. 213 to 652) per 100 000 people per year. The incidence for men was 1250 per 100 000 per year and for children 187 per 100 000 per year. None of the women reported onset of a groin hernia within the past year. A total of 10 001 household members were identified among the 1854 households. Among all the members of the participating households, 47 hernia repairs within the last year were reported by the household heads. This corresponds to 470 groin hernia repairs (95 per cent c.i. 350 to 620) per 100 000 people per year.

**Fig. 2:**
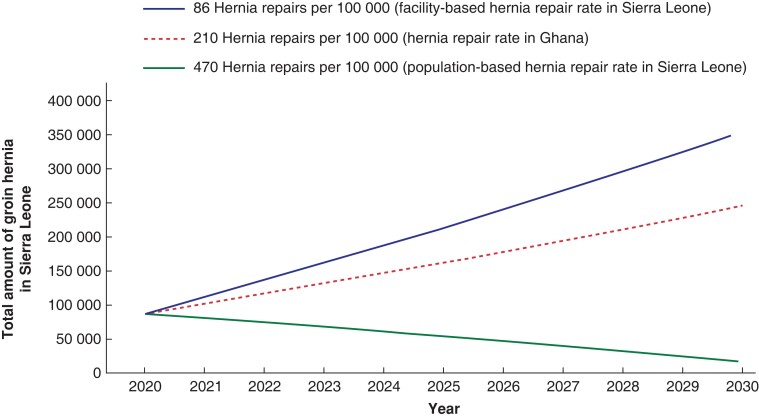
Three scenarios showing the effect of different groin hernia repair rates on prevalence of groin hernia among Sierra Leone people in 10 years All scenarios were corrected for an annual population growth of 2.14 per cent, which is equal to the population growth of Sierra Leone in 2018^22^.

Earlier findings suggest an increasing backlog of hernias in Sierra Leone. *Figure 2* shows three scenarios depending on different groin hernia repair rates given a prevalence of 1.1 per cent and an incidence of 389 groin hernias per 100 000 people, corresponding to 30 669 new groin hernias in Sierra Leone each year.

### Illness experience and health-seeking behaviour

Whereas 15 of the study participants with an untreated groin hernia (39) indicated no pain in the groin, another 12 experienced mild pain. For eight participants with a groin hernia, the pain was not ignorable (*Table 2*). Furthermore, 15 were not troubled by their hernia, 15 declared they were ashamed of their disease and eight were not able to work like they used to (*Table 3*). The main reason for not seeking healthcare or not undergoing surgery for a current groin hernia was the lack of financial resources (27), while two reported no need for healthcare. Less-frequently mentioned reasons were ‘fear or no trust in modern medicine’, ‘absence of healthcare providers within reach’, ‘pharmacological treatment only’, and ‘too young for surgery’. All participants with a present groin hernia reported they were aware of the possibility of having their condition surgically treated.

**Table 2:** Rate of inguinal pain as estimated by the participants with untreated groin hernia

Pain category	Participants *n* = 39
No pain	15
Pain present but can easily be ignored	12
Pain present, cannot be ignored but does not interfere with daily activities	3
Pain present, cannot be ignored and interferes with concentration and activities	1
Pain present, interferes with most activities	4
Pain present, necessitates bed rest	0
Pain present, prompt medical advice sought	0
Did not answer	4

Outcomes in this table are part of the validated Short-Form Inguinal Pain Questionnaire^28^.

**Table 3:** Daily life impact as estimated by the participants with untreated groin hernia

Impact on daily life	Participants *n* = 39
The condition is not disabling	15
I feel ashamed	15
I am not able to work like I used to	8
I need help with transportation	0
I need physical help with daily living	0
Did not answer	1

## Discussion

The prevalence of untreated groin hernia of 1.1 per cent seems to be lower compared with other studies from SSA^2–4,29^. Also within the three groups of men, women, and children, the groin hernia prevalence was found to be lower than reported earlier from various low-to-middle-income countries over the past decade^2–8,10,30^. The prevalence among men, whether it includes treated cases (6.3 per cent) or not (3.6 per cent), is clearly lower than reported in previous studies from SSA (9.4–13 per cent or 6.6–12.1 per cent)^2–6^. Three out of the five published papers on groin hernia epidemiology in SSA countries did not perform any kind of physical examination^2–4^. Two studies that performed physical examination on each participant and investigated rural populations only, reported a prevalence of untreated groin hernia among men of 6.6 per cent in Uganda and 10.8 per cent in Ghana, to be compared with 4.8 per cent among men in rural areas in this study^5,6^. The prevalence of untreated groin hernia among women and children was lower compared with previous findings in two studies that included verbal examination only and reported all kinds of groin masses^3,8^. A lower life expectancy in Sierra Leone compared with most other SSA countries might explain a lower hernia prevalence, as the risk of developing a groin hernia as an adult increases with age^31,32^. This and other studies indicate that groin hernias are associated with shame and other mental health strains, which might result in underreporting^3,33^.

A study from Uganda on children identified a discrepancy between self-reported groin hernia (0.4 per cent) and the prevalence verified by physical examination (1.4 per cent)^10^. It can be concluded that to achieve reliable results for groin hernia epidemiology, physical examination should be performed. In the present study, only study participants who reported that they had a groin hernia or mass were physically examined. Thus, some cases were probably missed, and consequently the groin hernia prevalence found in this study was possibly underestimated. The physical examination might also be more accurate when performed by surgeons instead of clinical assistants.

The prevalence of recurrent hernia in men was 14.5 per cent. In SSA, hernia repairs are typically performed without the use of mesh^34^. According to comprehensive data sets from high-income settings, long-term recurrence rates of well below 5 per cent are expected when using surgical mesh^35,36^. Reduced risk of reoperation is especially desirable for people with poor access to surgery, who for the same reason might have developed very large hernias that are complicated to repair. To reduce the risk of recurrence in this population, it would be desirable to introduce mesh hernia repair as routine practice. A recently published article randomizing mesh repair for inguinal hernia performed by medical doctors *versus* associate clinicians in Sierra Leone reported a 1-year recurrence rate of 3.5 per cent^37^. The study showed that both these types of surgical providers can perform hernia mesh repair safely and effectively and supports task sharing to reduce the backlog of hernias in Sierra Leone.

The incidence of 389 groin hernias per 100 000 people per year is higher than reported earlier in Africa, Europe, and the USA^12–16^, suggesting that groin hernias are as common, or more common in Sierra Leone, compared with many other countries. This incidence number should be interpreted with caution as groin hernias might be present but asymptomatic over time before someone notices its presence; however, this is not unique to Sierra Leone and is a methodological limitation affecting all the above studies.

The estimated repair rate of 470 out of 100 000 is surprisingly high and is likely an overestimation because of recall bias and misinterpretation by the household heads to classify operations as hernia repair. According to a study from 2014, hernia repair was the most common general surgical procedure in private and public hospitals in Sierra Leone nationwide, where a total rate of 86 repairs per 100 000 people was found^13^. A recent study interviewing surgical providers in Sierra Leone, described financial incentives not to record surgical activities in hospital logbooks, which again lead to systematic under-registration of surgical activity. In addition, there are several smaller, in many cases unlicensed, private clinics providing hernia surgery but, the extent and quality of their activities are unknown^38^.

The noticeable discrepancy between hernia repair rates retrieved from hospital logbooks *versus* the results of this household survey, deserves attention and requires further investigation. The true annual repair rate is likely somewhere in between the facility-reported figure and the current household study; however, towards which figure is hard to determine. This study supports, as demonstrated in *Fig. 2*, that the backlog of untreated groin hernias will remain for many years. If the annual repair rate is somewhere between 86 and 470 per 100 000, the backlog is more likely to increase over time, than decrease.

Conservative management is considered a safe approach for asymptomatic or minimally symptomatic inguinal hernias for men in high-income settings, and is unavoidably and widely practiced in Sierra Leone, as many cannot afford or prioritize costs for an operation^39^. Weak infrastructure makes follow-up-based strategies scarcely accessible, especially for the rural part of the population and therefore watchful waiting may not be appropriate. Assessment of correct diagnosis and proper selection of patients for surgery is crucial to avoid both unsuitable treatment and unnecessary burden from hernias left untreated. Elective surgery should be promoted to avoid risks associated with emergency hernia repair^40–42^.

In a setting where a lack of funds is the most common reason for failing to seek healthcare^3,5,6^, initiatives such as subsidized healthcare programmes and educational initiative towards all surgical providers on cost-effective and sustainable hernia management including introduction of low-cost mesh repairs, are keys to make quality treatment accessible and affordable and thereby reduce the backlog and burden of groin hernia. Uniform training of all surgical providers and continuing professional development are important to achieve proper quality of surgical care and to achieve low recurrence rate and beneficial long-term results. A large proportion of the hernia repairs cannot be accounted for in hospital statistics and are either not registered, or they are performed in private registered or unregistered clinics. Healthcare providers of any kind should be licensed and activity need to be supervised by the Sierra Leonean Medical and Dental Council.

There are multiple factors that might have had a favourable influence on the quality of this study. Participants who potentially had a groin hernia underwent physical examination by a CHO trained to provide surgical healthcare for groin hernias^20^. No groin hernias were diagnosed and noted without confirmation by one of the CHOs. Another strength is that the enumerators had a medical background or experience in performing medically related interviews, and they attended a 1-week training session covering each aspect of PRESSCO 2020. Data were checked on missing and incorrect elements both in the field and by the core members of the research group on the same day the data were collected. This real-time feedback enabled enumerators to correct errors detected.

A limitation of this study is the absence of an internal validation of the method. Study participants that mentioned potential groin pathology consequently underwent a physical examination by one of the CHOs, whereas those not mentioning potential groin pathology were not examined; however, a hernia not known to an individual, may not deserve attention, or may be undetectable for a clinician as well. As in general with interview-based research, reporting bias associated with cultural aspects, such as male participants being interviewed by female enumerators and vice versa, may affect the results. False recollections might have introduced a recall bias, possibly affecting prevalence and incidence numbers as well as repair rates. As hernia repair is among one of the most common surgical operations, other operations could be misclassified as a hernia repair, contributing to an overestimation of the repair rate. Furthermore, the questionnaire was written in English, whereas many other languages are spoken all over Sierra Leone. Translation and interpretation of words may differ between the enumerator and the study participant but even between the enumerators themselves, possibly weakening the quality of data.

Groin hernia is a common condition in Sierra Leone, although this study found the prevalence to be lower than previously reported in SSA. The existing backlog of untreated groin hernias is likely to remain, as the incidence and the repair rates are similar. Inability to pay is a common reason to not seek healthcare for a groin hernia. Possibilities for further development of groin hernia care in Sierra Leone might lay within better surgical attention in rural areas by promoting surgical task sharing, lowering the recurrence rate by introducing low-cost meshes, and tackling the financial barriers to treatment.

## Data Availability

The data set of this study is available upon request by contacting the corresponding author. The authors confirm that the data supporting the findings of this study are available within the article and its supplementary materials.
